# Dual tachycardia in a premature infant: a rare case report and literature review

**DOI:** 10.3389/fcvm.2025.1712012

**Published:** 2026-01-21

**Authors:** Meng Xu, Qingzhu Qiu, Chongbing Yan, Zhen Yan, Cuilan Hou, Tingting Xiao, Li Zhang

**Affiliations:** 1 Department of Cardiology, Shanghai Children’s Hospital, School of Medicine, Shanghai Jiao Tong University, Shanghai, China; 2 Department of Neonatology, Shanghai Children’s Hospital, School of Medicine, Shanghai Jiao Tong University, Shanghai, China

**Keywords:** dual tachycardia, esophageal electrocardiogram, persistent atrial tachycardia, premature infant, ventricular tachycardia

## Abstract

**Objective:**

To enhance understanding of the diagnosis and management of dual tachycardia in infant.

**Methods:**

A retrospective analysis was conducted on the clinical data and management of a infant with dual tachycardia. A review of the relevant literature was also performed.

**Results:**

A female infant, born 43 min, was transferred to our hospital's NICU via emergency transfer due to “tachycardia lasting over half an hour after premature birth.” To better understand the supraventricular tachycardia an esophageal electrode was inserted, the esophageal electrocardiogram confirmed the diagnosis of dual tachycardia (persistent atrial tachycardia combined with short episodes of ventricular tachycardia).

**Conclusion:**

This case provides valuable insight into the diagnosis and management of dual tachycardia in infant. For patients presenting with tachycardia, esophageal electrocardiogram is crucial.

## Introduction

The normal fetal heart rhythm is regular, with a heart rate range of 110–160 beats per minute. Fetal tachycardia is defined as an increased fetal heart rate exceeding 180 beats per minute (1). Fetal tachyarrhythmias are more common in the later stages of pregnancy, making up about 0.5% of all fetal arrhythmias (2). Types of fetal tachyarrhythmias such as sinus tachycardia, paroxysmal supraventricular tachycardia, atrial tachycardia, atrial flutter, atrial fibrillation, and ventricular tachycardia are included (3). The case of a female infant born at 32 weeks and 4 days gestation, experiencing persistent tachycardia at 180–210 beats per minute shortly after birth, serves as a critical illustration of dual tachycardia in neonates. The diagnostic challenge was further complicated by the presence of both supraventricular and wide QRS complex tachycardias, necessitating the use of an esophageal electrode to precisely differentiate between the arrhythmias. The clinical significance of this case lies in its status as the first reported instance of dual tachycardia in a neonate, thus contributing novel insights into the understanding of neonatal heart rhythm disorders.

## Case description

**Figure 1:**
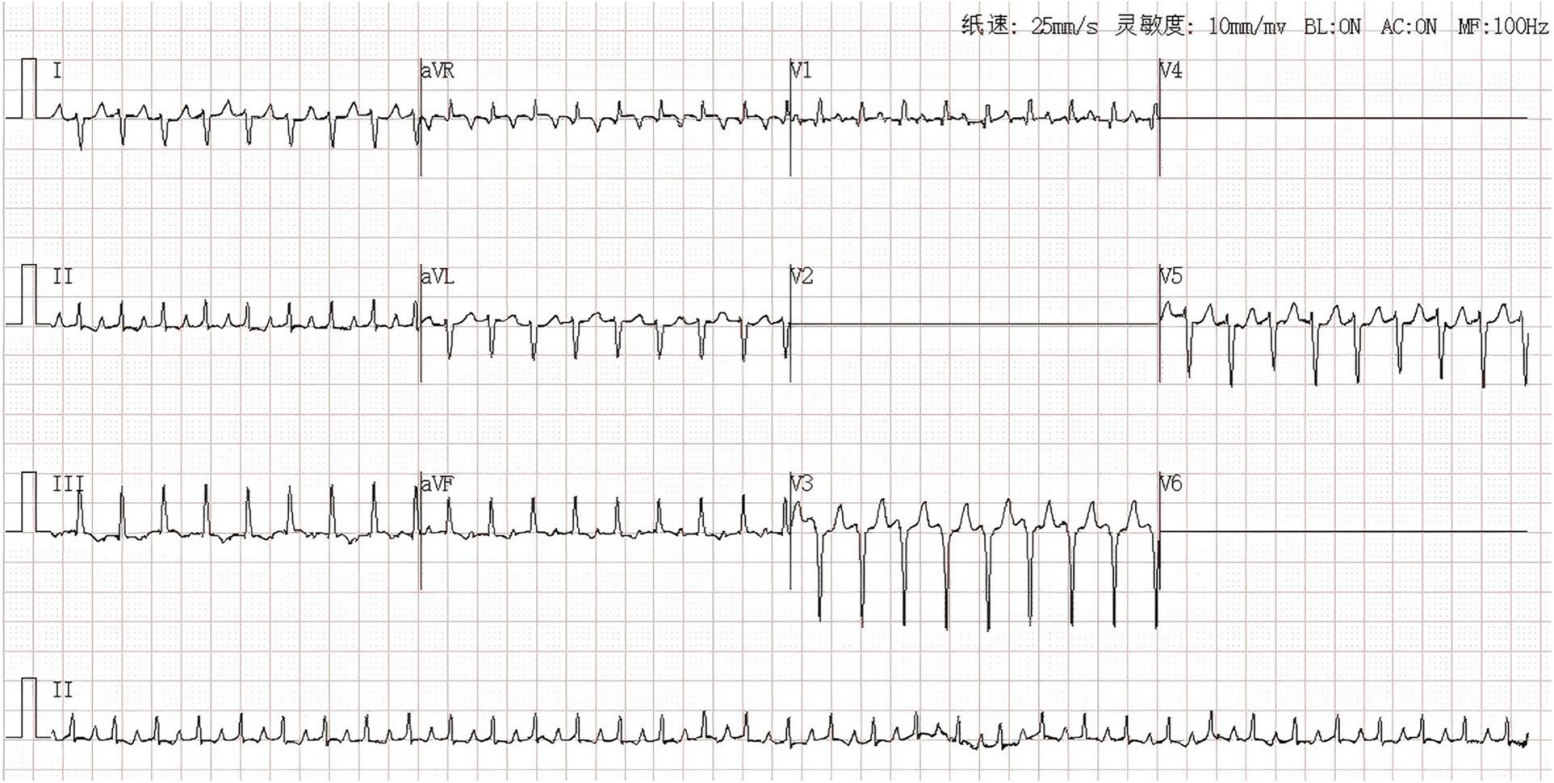
Narrow QRS complex tachycardia: ventricular rate 210 bpm, QRS duration 64 ms, QRS axis 126.

A female infant, born 43 min, was transferred to our hospital's NICU via emergency transfer due to “tachycardia lasting over half an hour after premature birth.” Three days prior to birth, fetal heart monitoring indicated a baseline fetal heart rate of 180 beats per minute. On the day of birth, fetal echocardiography showed an enlarged cardiothoracic ratio, a dilated right ventricle, moderate tricuspid regurgitation, and fetal tachycardia; there was also fluid accumulation in the right thoracic and abdominal cavities. The delivery hospital diagnosed “fetal edema and arrhythmia” and performed an emergency cesarean section. The infant born at 32 + 4 weeks gestation, with a birth weight of 2,250 g, was the fourth child of the mother who had two previous pregnancies. The amniotic fluid was clear, and there were no abnormalities with the placenta or umbilical cord. The Apgar scores were 8 at 1 min and 9 at 5 min, with a postnatal heart rate fluctuating between 180 and 210 beats per minute. After being transferred to NICU, the infant's preterm assessment score was 15, equivalent to a gestational age of 34 + 5 weeks. Due to respiratory distress, manifested as tachypnea and retractions (+), and with spontaneous breathing, the infant received continuous positive airway pressure ventilation. Breath sounds were symmetrical and coarse, and no rales were detected; heart rate was 210 beats per minute, with strong heart sounds and a regular rhythm, and no murmurs were noted; the abdomen felt soft and wasn't distended; bilateral lower limb edema (+); right upper limb oxygen saturation was 92%, and left lower limb oxygen saturation was 93%. The admission electrocardiogram showed narrow QRS complex tachycardia, with a ventricular rate of 210 bpm, QRS duration of 64 ms, and QRS axis of 126° (Figure 1). Echocardiography showed a secundum-type atrial septal defect (0.37 cm, left-to-right shunt), patent ductus arteriosus (0.3 cm, bidirectional shunt), pulmonary hypertension, and reduced left ventricular systolic function (EF = 42.2%). Laboratory tests showed: CK-MB 419 U/L (the normal reference range is 0–25 U/L), cTnI 0.039 ng/mL (the normal reference range is 0–0.026 ng/mL), BNP 2,499.24 pg/mL (the normal reference range is 0–100.00 pg/mL). The initial diagnoses included neonatal arrhythmia (supraventricular tachycardia, SVT), heart failure, and congenital heart disease. They administered vagal stimulation and a 0.2 mg bolus of adenosine twice, reducing the ventricular rate to 180 beats/min; however, supraventricular tachycardia persisted. Furthermore, continuous ECG monitoring revealed short runs of wide QRS complex tachycardia.

To better understand the supraventricular tachycardia and to determine if the wide QRS complex tachycardia was caused by supraventricular tachycardia with intraventricular conduction delay or by ventricular tachycardia, an esophageal electrode was inserted for esophageal electrocardiogram examination and to terminate an episode of SVT.

**Figure 2:**
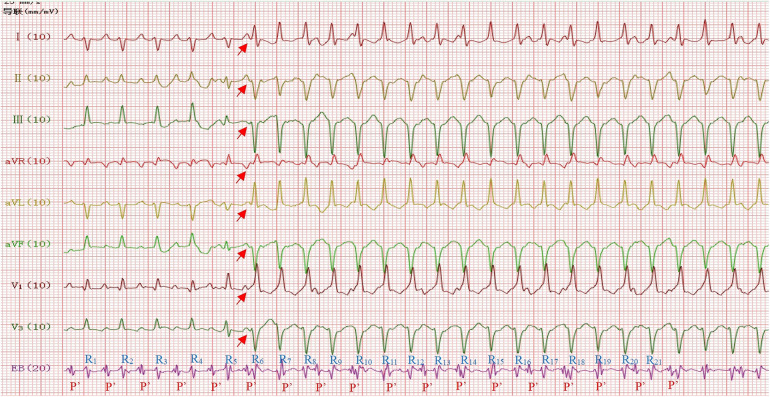
Narrow QRS wave tachycardia: ventricular rate 182 beats/min (RR interval = 328 ms), P’P ‘interval = 328 ms, RP’interval = 176 ms, P’R interval = 152 ms, P’upright in leads I, II, III, aVF,V1,V3 and P’inversion in lead aVR (Red arrow). Wide QRS wave tachycardia: ventricular rate 241 beats/min (RR interval = 248 ms), QRS duration 74 ms, QRS electrical axis −83°, R5 was identified as a ventricular fusion wave. EB = Esophageal Bipolar Lead.

**Figure 3:**
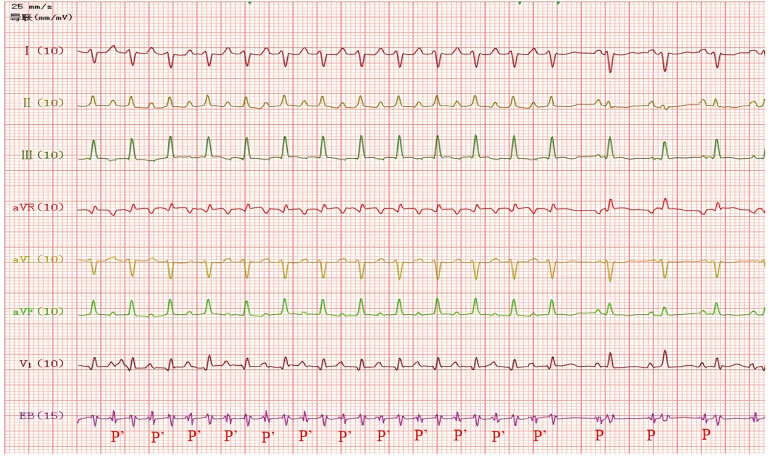
Narrow QRS tachycardia converted to sinus rhythm, ventricular rate 136 beats/min, PR interval = 110 ms.

Using the DF-5A cardiac electrophysiological diagnostic and therapeutic instrument produced by Suzhou Oriental Electronic Instrument Factory, a 4-pole esophageal electrode catheter was inserted through the mouth and advanced to 15 cm from the teeth, where the largest atrial wave was recorded. Continuous recordings from the body surface and esophageal ECG showed alternating narrow and wide QRS tachycardia (Figure 2). During narrow QRS wave tachycardia, the ventricular rate was 182 beats per minute (RR interval = 328 ms), with the esophageal lead showing a clear and prominent P' wave that appeared multi-directional, with a P'P' interval of 328 ms, an RP' interval of 176 ms, and a P'R interval of 152 ms. The RP' interval was greater than the P'R interval, which indicated that the P'R interval was prolonged, with the P' wave upright in leads I, II, III, and aVF. During wide QRS wave tachycardia, the ventricular rate was 241 beats per minute (RR interval = 248 ms), with a QRS duration of 74 ms and a QRS axis of −83°. The V_1_ and aVR leads showed a unidirectional R wave pattern, while the II, III, and aVF leads exhibited an rS pattern, with S_III_ > S_II_, suggesting a left anterior fascicular block. The morphology of the P' wave and the P'P' interval were the same as during narrow QRS wave tachycardia, with the ventricular rate exceeding the atrial rate and atrioventricular dissociation, suggesting ventricular tachycardia, where R_5_ was identified as a ventricular fusion wave. During the recording of the esophageal electrocardiogram, the supraventricular tachycardia stopped on its own, restoring sinus rhythm, with a normal PR interval. Through careful comparative analysis, the P wave in lead III is inverted during narrow QRS complex tachycardia, while it is upright during sinus rhythm (Figure 3). Therefore, the esophageal electrocardiogram confirmed the diagnosis of dual tachycardia (persistent atrial tachycardia combined with short episodes of ventricular tachycardia).

### Clinical course

After identifying the type of rapid arrhythmia, the patient received digoxin 10 µg orally every 12 h, along with fluid restrictions, diuretics, and support with plasma and albumin, epinephrine for cardiac strengthening, dopamine to improve microcirculation, and ventilatory support as part of comprehensive treatment. From the fourth day of hospitalization, the patient's atrial tachycardia ceased, and the dominant rhythm returned to sinus rhythm. The next day, a follow-up echocardiogram showed normal left ventricular systolic function (EF 61%). A 24-hour dynamic ECG examination after 13 days of hospitalization revealed 1,520 premature ventricular contractions, 53 pairs of premature ventricular contractions, and 1 short episode of ventricular tachycardia (frequency 202 bpm, monomorphic); there were 21 premature atrial contractions. A follow-up dynamic ECG after two months was normal.

## Discussion

The hemodynamic effects of fetal tachyarrhythmias include low cardiac output, increased central venous pressure, which can lead to fetal edema, progressive fetal placental circulation failure, and fetal death (4–6). If fluid retention occurs in two or more fetal cavities, it is important to consider the possibility of edema (4, 7). This baby was delivered via emergency cesarean section because of fetal tachycardia and edema. After birth, ECG monitoring showed supraventricular tachycardia, followed by intermittent narrow and wide QRS complex tachycardia. Continuous recording of the esophageal electrocardiogram combined with the surface electrocardiogram confirmed the diagnosis of persistent atrial tachycardia combined with short episodes of idiopathic ventricular tachycardia. These two types of tachyarrhythmias in newborns are rare and represent a dual tachycardia, reported for the first time. Currently, only two cases have been reported in adults (8, 9). One case was a 40-year-old female with atrial fibrillation and ventricular tachycardia. The other case was a 20-year-old male with sinus tachycardia and junctional tachycardia.

Dual heart rhythm refers to a heart rhythm formed by two different pacing points simultaneously sending impulses that control different parts of the heart. Each ectopic pacing point emits abnormally rapid impulses that control a portion of the heart. When both occur simultaneously within the same cardiac cycle, it is termed dual tachycardia; when they alternate in sequence, it is referred to as alternating tachycardia. The initial routine electrocardiogram taken upon the child's admission showed regular narrow QRS wave tachycardia, with no P waves seen before or after the QRS wave, leading to a diagnosis of supraventricular tachycardia. Esophageal ECG combined with surface electrocardiography can clearly display the atrial rhythm, showing a 1:1 conduction ratio between the atria and ventricles during supraventricular tachycardia. During wide QRS wave tachycardia, the P' P' interval can be accurately measured, indicating ventricular tachycardia and ruling out supraventricular tachycardia with intraventricular conduction differences. The morphology of the P' wave and the P’ P' interval stay consistent during both narrow and wide QRS wave tachycardias, suggesting that the atrial rhythm in both types of tachycardia is controlled by the same pacing point. This case does not present with alternating episodes of atrial tachycardia and ventricular tachycardia, but rather with dual tachycardia. Combined with the distinct morphology of P waves in lead III during narrow QRS tachycardia vs. sinus rhythm (with normal PR interval in sinus rhythm), the diagnosis of atrial tachycardia is confirmed. Occasionally, the electrocardiographic manifestations of atrial tachycardia may resemble sinus tachycardia, necessitating careful differentiation (10). In this case, during dual tachycardia, the two ectopic pacing points control the atrial and ventricular rhythms respectively, but it's still unclear if these two types of tachycardia are connected.

The occurrence of atrial tachycardia in newborns is closely related to changes in atrial electrophysiological characteristics, the presence of undifferentiated autonomic cells or reentrant circuits within the atrium (11, 12). Ectopic atrial tachycardia is associated with increased atrial automaticity, characterized by abnormal P waves accompanied by persistent tachycardia (13). The most common ectopic foci are located in the right atrial appendage, crista terminalis, and around the pulmonary veins, but foci can exist in any location within the right or left atrium (14). This type of tachycardia might not respond to adenosine or direct current cardioversion, and if not managed well, it can cause tachycardia-induced cardiomyopathy (13). Medications are the primary method for treating arrhythmias in newborns. Currently, there is no consensus or evidence regarding the best indications for medication use or the duration of therapy, and there aren't many clinical trials on drug treatments (15–17). In this case, stimulation of the vagus nerve and intravenous adenosine injection failed to terminate the atrial tachycardia. Digoxin has the effect of increasing myocardial contractility while also slowing the heart rate and delaying atrioventricular conduction. Given the child's heart failure, oral digoxin treatment was given. In some centers, it is the preferred medication for infant supraventricular tachycardia (16, 18, 19) and has historically been the most commonly used first-line drug for treating fetal tachycardia (7), with a success rate between 46% and 62%. Three days after oral digoxin treatment, the atrial tachycardia reverted to sinus rhythm, and ventricular contraction function returned to normal, showing effective treatment. For wide QRS complex tachycardia, doctors use esophageal electrocardiography to tell what kind of tachycardia it is, and esophageal pacing is not recommended as a method to terminate ventricular tachycardia (20). Generally, drug treatment is used for hemodynamically unstable ventricular tachycardia. In this case, the short episodes of ventricular tachycardia went away on their own two months after birth without antiarrhythmic drugs. Most newborns with ventricular tachycardia do well and usually get better on their own within a few months after birth (21–24).

The overlapped P waves in surface electrocardiograms pose challenges for diagnosis and classification in cases of atrial tachycardia in newborns (25). Esophageal electrocardiography can capture tall, clear P waves, clarifying atrial-ventricular relationships and helping to identify arrhythmias that are hard to see on surface ECGs.

Esophageal atrial pacing can be performed at the bedside with minimal complications. It has been effective in diagnosing and restoring rhythm in newborns with fast arrhythmias (26). Atrial tachycardia is relatively rare in the neonatal period, while ventricular tachycardia is even rarer; this is the first reported case of dual tachycardia. Once the type of arrhythmia is clarified, targeted treatments can be chosen to quickly restore sinus rhythm.

## Data Availability

The raw data supporting the conclusions of this article will be made available by the authors, without undue reservation.
